# Structural Connectivity Gradients of the Temporal Lobe Serve as Multiscale Axes of Brain Organization and Cortical Evolution

**DOI:** 10.1093/cercor/bhab149

**Published:** 2021-06-19

**Authors:** Reinder Vos de Wael, Jessica Royer, Shahin Tavakol, Yezhou Wang, Casey Paquola, Oualid Benkarim, Nicole Eichert, Sara Larivière, Ting Xu, Bratislav Misic, Jonathan Smallwood, Sofie L Valk, Boris C Bernhardt

**Affiliations:** McConnell Brain Imaging Centre, Montreal Neurological Institute, McGill University, Montreal, Quebec, H3A 2B4, Canada; McConnell Brain Imaging Centre, Montreal Neurological Institute, McGill University, Montreal, Quebec, H3A 2B4, Canada; McConnell Brain Imaging Centre, Montreal Neurological Institute, McGill University, Montreal, Quebec, H3A 2B4, Canada; McConnell Brain Imaging Centre, Montreal Neurological Institute, McGill University, Montreal, Quebec, H3A 2B4, Canada; McConnell Brain Imaging Centre, Montreal Neurological Institute, McGill University, Montreal, Quebec, H3A 2B4, Canada; McConnell Brain Imaging Centre, Montreal Neurological Institute, McGill University, Montreal, Quebec, H3A 2B4, Canada; Wellcome Centre for Integrative Neuroimaging, Nuffield Department of Clinical Neurosciences, John Radcliffe Hospital, University of Oxford, Oxford, OX3 9DU, UK; McConnell Brain Imaging Centre, Montreal Neurological Institute, McGill University, Montreal, Quebec, H3A 2B4, Canada; Center for the Developing Brain, Child Mind Institute, New York, NY, NY 10022, USA; McConnell Brain Imaging Centre, Montreal Neurological Institute, McGill University, Montreal, Quebec, H3A 2B4, Canada; Queens University, Kingston, Ontario, K7L 3N6, Canada; Max Planck Institute for Human Cognitive and Brain Sciences, Leipzig, 04103, Germany; McConnell Brain Imaging Centre, Montreal Neurological Institute, McGill University, Montreal, Quebec, H3A 2B4, Canada

**Keywords:** connectome, gradients, MRI, multimodal, neuroimaging

## Abstract

The temporal lobe is implicated in higher cognitive processes and is one of the regions that underwent substantial reorganization during primate evolution. Its functions are instantiated, in part, by the complex layout of its structural connections. Here, we identified low-dimensional representations of structural connectivity variations in human temporal cortex and explored their microstructural underpinnings and associations to macroscale function. We identified three eigenmodes which described gradients in structural connectivity. These gradients reflected inter-regional variations in cortical microstructure derived from quantitative magnetic resonance imaging and postmortem histology. Gradient-informed models accurately predicted macroscale measures of temporal lobe function. Furthermore, the identified gradients aligned closely with established measures of functional reconfiguration and areal expansion between macaques and humans, highlighting their potential role in shaping temporal lobe function throughout primate evolution. Findings were replicated in several datasets. Our results provide robust evidence for three axes of structural connectivity in human temporal cortex with consistent microstructural underpinnings and contributions to large-scale brain network function.

## Introduction

The human temporal lobe is involved in multiple cognitive, affective, and sensory processes, including memory (Vaz et al. 2019), emotional reactivity (Phelps 2004), semantic cognition (Ralph et al. 2017), as well as auditory processing (Bonilha et al. 2017). Notably, temporal lobe subregions have been suggested to serve as origins of major organizational and evolutionary axes of the human brain (Sanides 1969; Goulas et al. 2019), and host structures, such as the middle and superior temporal gyri, that have undergone accelerated functional reconfigurations and areal expansion in primate evolution (Mars, Sotiropoulos, et al. 2018; Eichert et al. 2020; Xu et al. 2020). Collectively, these different aspects suggest that the temporal lobe is a hub implicated in important features of human cognition, and that its study may provide key insights into cortical organization and its phylogenetic basis.

In an attempt to understand the role of the temporal lobe in whole-brain networks, prior studies in nonhuman animals as well as humans have started to delineate the complex connectivity profiles of the temporal lobe. Tract tracing studies in nonhuman primates have charted short range connections as well as long-range tracts of the temporal lobe (Webster et al. 1991), showing distributed connectivity patterns to a diverse territory of cytoarchitectural areas (Morán et al. 1987; Mohedano-Moriano et al. 2015; Beul et al. 2017; Sakata et al. 2019). These findings were complemented by diffusion magnetic resonance imaging (MRI) tractography studies in both nonhuman primates (Bryant et al. 2020) and humans (Saur et al. 2008), where this noninvasive technique can approximate the course of white matter fiber tracts both in vivo and ex vivo. Diffusion MRI studies have been performed for all major long range fiber bundles (Smiley and Falchier 2009; Howells et al. 2018; Roumazeilles et al. 2020), for short-range fiber systems (Attar et al. 2020) as well as the superficial white matter (Oishi et al. 2008; Liu et al. 2016; Hong, Hyung, et al. 2019; Bodin et al. 2020). Using resting-state functional MRI, regions of the temporal lobe have been implicated in several major networks, particularly the default mode, limbic, and visual networks (Yeo et al. 2011). Cross-species studies have found phylogenetic divergences of connectivity and cortical area between humans and macaques. Specifically, the lateral temporal cortex is among the areas that have the most diverging connectivity profile and have undergone the most marked cortical expansion in humans relative to macaques, conversely the area of the medial temporal cortex and its functional connectivity are relatively preserved across species (Xu et al. 2020). Despite parallel advances in our understanding of the temporal lobe across these modalities, there is a lack of integration of these findings into a cohesive model of temporal lobe organization.

Beyond the mapping of specific connectivity changes, recent years have seen a shift towards the application of unsupervised approaches that identify and visualize low-dimensional eigenmodes in connectivity changes across the cortical mantle—also referred to as connectivity gradients (Margulies et al. 2016; Huntenburg et al. 2018). A gradient perspective describes transitions of brain connectivity in a continuous reference frame, which has been proposed to capture subregional heterogeneity as well as functional multiplicity better than techniques that parcellate cortex into discrete subregions and average potentially variable properties within parcels (Mars, Sotiropoulos, et al. 2018; Bijsterbosch et al. 2020; Haak and Beckmann 2020). Capitalizing on resting-state functional MRI acquisitions, gradient mapping techniques have previously identified principal axes of neural organization in healthy adults and in nonhuman primates (Margulies et al. 2016; Guell et al. 2018; Haak et al. 2018; Vos de Wael et al. 2018; Buckner and Margulies 2019; Xu et al. 2020), and these techniques are increasingly used to study lifespan processes related to aging (Lowe et al. 2019; Bethlehem et al. 2020) and typical as well as atypical childhood development (Hong, Vos de Wael, et al. 2019; Larivière et al. 2019; Ball, Seidlitz, Beare, et al. 2020; Ball, Seidlitz, O’Muircheartaigh, et al. 2020; Park, Hong, et al. 2020). By reducing high-dimensional connectivity data to a few eigenvectors that describe spatial axes of connectivity variations, these techniques allow for a contextualization with typical spatial features of neural organization, including cortical thickness measures (Larivière et al. 2019), intracortical myelin estimates (Huntenburg et al. 2017), and task-derived functional activation patterns (Murphy et al. 2019). In the temporal lobe, these techniques have previously been applied to structural connectivity information, with the goal of subsequent parcellation (Bajada et al. 2017), to describe the ventral and anterior temporal lobe as a structural connectivity convergence zone (Bajada et al. 2019), and to relate structural connectivity gradients to meta-analytic task activations (Yarkoni et al. 2011; Blazquez Freches et al. 2020).

In the current work, we expanded on these previous findings in three ways:

(i) We explored regional associations between structural connectivity gradients in the temporal lobe and measures of intracortical microstructure to assess whether large scale connectivity axes are reflected in the local microcircuits. Prior findings in nonhuman animals suggest that an area’s cytoarchitectonic properties may be predictive of structural connectivity, but precise associations between both remain underspecified in humans (Barbas 2015). To fill this gap, our project leveraged both myelin sensitive MRI contrasts as well as postmortem cytoarchitecture analysis based on BigBrain (Amunts et al. 2013).
(ii) Structural connectivity is generally assumed to constrain functional connectivity (Honey et al. 2009; Deco et al. 2017; Wang et al. 2019; Suárez et al. 2020). Here, we assessed whether structural connectome gradients within the temporal lobe, as a low-dimensional representation of structural connectivity, can predict intrinsic functional organization derived from resting-state functional magnetic resonance imaging (fMRI) acquisitions, both with respect to macroscale functional motifs as well as node-wise estimates of functional connectivity.
(iii) Finally, to determine phylogenetic principles of structural connectome organization, we examined whether structural connectivity gradients reflect principal dimensions of primate evolution. To this end, we studied the relationship of gradients with areal expansion and functional reconfigurations from nonhuman primates to humans (Xu et al. 2020).

Our approach capitalized on multimodal image processing and advanced diffusion tractography analyses. Specifically, we leveraged a high-resolution representation of temporal lobe structural connectivity to resolve subregional heterogeneity in connectivity and multiplicity of potentially overlapping gradients. Our findings were replicated both in a hold-out dataset from the same site, and in a dataset acquired with a different scanner, imaging parameters, and preprocessing pipeline. We have released all codes to replicate the main figures on https://github.com/MICA-MNI/micaopen.

## Materials and Methods

### Overview

To foreshadow our approach (for details see the remainder of the Methods), we computed vertex-wise structural connectivity from the temporal lobe to the rest of the brain in three independent datasets. Diffusion map embedding decomposed structural connectomes in each dataset into eigenvectors describing spatial gradients of connectivity. We first assessed spatial associations to other structural connectome features (i.e., connectivity distance and degree centrality). Secondly, we investigated associations to curvature, cortical thickness, and estimates of cortical myelin. Third, we predicted motifs of resting-state functional connectivity from the structural connectome gradients. Lastly, we assessed relations to phylogenetic markers (Xu et al. 2020). Spatial association analyses controlled for autocorrelations using Moran’s spectral randomization.

### Participants

We selected 150 unrelated participants from the Human Connectome Project (HCP) dataset for whom all resting-state, diffusion weighted imaging, and structural scans were available and completed in full (Van Essen et al. 2013). These participants were split into HCP-Discovery (*n* = 75; age = 29.2 ± 3.6, female = 47) and HCP-Replication (*n* = 75; age = 28.9 ± 4.0, female = 44) datasets. For the microstructure informed connectomics (MICs) dataset, all data were collected in a single testing session per participant between April 2018 and March 2020. Participants (*n* = 54; 30.5 ± 7.3, female = 20) all provided informed consent. Participants reported no history of neurological illness. The study was approved by the Ethics Committee of the Montreal Neurological Institute and Hospital.

### Image Acquisition

(a) HCP. Images were acquired on the customized Siemens 3 T “Connectome Skyra.” Two T1w images were acquired with a 3D MPRAGE sequence with the following parameters: TR = 2400 ms, TE = 2.14 ms, flip angle = 8 deg, FOV = 224 x 224 mm^2^, voxel size = 0.7 mm isotropic. Two T2w images were acquired with identical parameters except for the following: TR = 3200 ms, TE = 565 ms, variable flip angle. Four resting-state fMRI images were acquired with a gradient-echo echo-planar imaging (EPI) sequence (TR = 720 ms, TE = 33.1 ms, flip angle = 52 deg, FOV = 208 x 180 mm, 2 mm isotropic voxels, and 1200 volumes per run). Diffusion images were acquired with a spin-echo EPI sequence (TR = 5520 ms, TE = 89.5 ms, flip angle = 78 deg, FOV = 210 x 180 mm, 1.25 mm isotropic voxels, *b*-values 1000, 2000, and 3000 s/mm^2^, 90 diffusion-weighting directions). Six diffusion image scans were acquired each lasting 9 min and 50 s. Half the runs were acquired with left-to-right phase encoding and the other half with right-to-left.

(b) MICs. Images were acquired on a 3 T Siemens Magnetom Prisma-Fit equipped with a 64-channel head coil. Two T1w scans were acquired with a 3D-MPRAGE sequence (0.8 mm isotropic voxels, matrix = 320 × 320, 224 sagittal slices, TR = 2300 ms, TE = 3.14 ms, TI = 900 ms, flip angle = 9°, iPAT = 2). Quantitative T1 (qT1) relaxometry data were acquired using a 3D-MP2RAGE sequence (0.8 mm isotropic voxels, 240 sagittal slices, TR = 5000 ms, TE = 2.9 ms, TI 1 = 940 ms, T1 2 = 2830 ms, flip angle 1 = 4°, flip angle 2 = 5°, iPAT = 3, bandwidth = 270 Hz/px, echo spacing = 7.2 ms, partial Fourier = 6/8). We combined two inversion images for qT1 mapping to minimize sensitivity to B1 inhomogeneities and optimize intra- and inter-subject reliability (Marques et al. 2010; Haast et al. 2016). DWI images were obtained with spin-echo EPI, including three shells with *b*-values 300, 700, and 2000s/mm^2^ and 10, 40, and 90 diffusion weighting directions per shell, respectively (TR = 3500 ms, TE = 64.40 ms, 1.6 mm isotropic voxels, flip angle = 90°, refocusing flip angle = 180°, FOV = 224 × 224 mm^2^, slice thickness = 1.6 mm, multiband factor = 3, echo spacing = 0.76 ms, number of b0 images = 3). One 7 min rs-fMRI scan was acquired using multiband accelerated 2D-BOLD EPI (TR = 600 ms, TE = 30 ms, 3 mm isotropic voxels, flip angle = 52°, FOV = 240 × 240 mm^2^, slice thickness = 3 mm, multiband factor = 6, echo spacing = 0.54 ms). Participants were instructed to keep their eyes open, look at a fixation cross, and not fall asleep. Two spin-echo images with reverse phase encoding were acquired for distortion correction of the rsfMRI scans (phase encoding = AP/PA, 3 mm isotropic voxels, FOV = 240 × 240 mm^2^, slice thickness = 3 mm, TR = 4029 ms, TE = 48 ms, flip angle = 90°, echo spacing = 0.54 ms, bandwidth = 2084 Hz/Px).

### Structural Preprocessing

(a) HCP. Structural images underwent standard HCP preprocessing (Glasser et al. 2013). In short, T1w images were corrected for gradient nonlinearity. Repeated scans were coregistered and averaged. After brain extraction and readout distortion correction, T1w and T2w images were co-registered using rigid body transformations. Nonuniformity correction based on the T1w and T2w contrasts was applied. Preprocessed images were nonlinearly registered to MNI152 space. Cortical surfaces were extracted using FreeSurfer 5.3.0-HCP (Dale et al. 1999; Fischl, Sereno, and Dale 1999; Fischl, Sereno, Tootell, et al. 1999), with minor modifications to incorporate information from both T1w and T2w scans. Cortical surfaces were aligned using MSMAll to the Conte69 template (Robinson et al. 2014).

(b) MICs. Data were preprocessed with a Freesurfer 6.0 recon_all pipeline. Both native T1w scans were provided as input and combined through this pipeline. Manual corrections of the pial and white matter surfaces were performed for all subjects. Curvature and cortical thickness estimates were generated by the recon_all pipeline. To acquire tissue segmentations for anatomically constrained tractography, the same images underwent a separate pipeline which included linear alignment of both T1w scans, nonuniformity correction, and intensity normalization. Corrected images were segmented into tissue types using MRtrix3’s 5ttgen (Smith et al. 2012). qT1 images were linearly aligned to the cortical surface using boundary-based registration (Greve and Fischl 2009). qT1 values were interpolated to the surface by taking the average of seven trilinear interpolations evenly interspersed between the 20th and 80th percentile distances from the pial to white matter surfaces using Freesurfer’s *mri_vol2surf* command.

### Resting-State Preprocessing

(a) HCP. Data underwent standard HCP preprocessing (Glasser et al. 2013). In short, the timeseries were corrected for gradient nonlinearity and head-motion. The R-L/L-R scan pairs were used to correct for geometric distortions. Resulting images were warped to the structural image using rigid body and boundary-based registrations. This warp was concatenated with the warp from T1w image space to MNI152 space to transform functional images to MNI152 space. Further processing removed the bias field, removed the skull, and normalized whole brain intensity. A high pass filter (>2000s FWHM) was used to correct for scanner drift, and additional noise was removed using ICA-FIX (Salimi-Khorshidi et al. 2014).

(b) MICs. The first five volumes were discarded to allow for magnetic field saturation. Images were then reoriented, and motion and distortion corrected. Nuisance variable signal was removed using an ICA-FIX classifier trained on this dataset and subsequent spike regression (Salimi-Khorshidi et al. 2014). Further tissue-specific signal regression was not performed (Murphy and Fox 2017; Vos de Wael et al. 2017). A warp to the Freesurfer T1w image was computed by averaging volumetric timeseries across the time dimension and registering this image using boundary-based registration. Timeseries were sampled to the surface by taking the average of seven trilinear interpolations evenly interspersed between the 20th and 80th percentile distances from the pial to white matter surfaces.

### Diffusion Preprocessing

(a) HCP. Images underwent standard HCP preprocessing (Glasser et al. 2013). In short, image intensity was normalized across scans. The topup and eddy algorithms were used to correct for EPI distortions, eddy currents, and motion. A gradient nonlinearity correction was performed, and the deviation of the *b*-values and *b*-vectors was computed. Mean b0 images were registered to the T1w image with boundary-based registration (Greve and Fischl 2009), and this registration was used to transform DWI images to T1w space. The brain was masked based on a Freesurfer segmentation.

(b) MICs. Data were preprocessed and denoised with MRTrix3’s dwipreproc, which is based on FSL’s eddy correction and topup, and dwidenoise (Andersson et al. 2003; Smith et al. 2004; Tournier et al. 2012). Freesurfer segmentations were registered to the subject’s DWI space using boundary-based registration (Greve and Fischl 2009).

### High-Resolution Diffusion Tractography and Gradient Mapping

Tractography was performed identically for the HCP and MICs dataset with MrTrix3 (Tournier et al. 2012). Response functions for each tissue type were estimated using the dhollander algorithm (Dhollander et al. 2016). Fiber orientation distributions were modeled with multishell multitissue spherical deconvolution (Jeurissen et al. 2014) and subsequently underwent multitissue informed log-domain intensity normalization. The structural T1w image was segmented into five tissue types (Smith et al. 2012). Anatomically constrained tractography was performed systematically for each temporal lobe voxel in the gray–white matter interface by generating streamlines using second order integration over fiber orientation distributions (Tournier et al. 2010). Streamlines were seeded dynamically from the white matter using the SIFT model (Smith et al. 2015a, 2015b). Streamline generation was aborted when 40 million streamlines had been accepted. Each streamline was assigned a weight by optimizing a cross-section multiplier derived with the SIFT2 algorithm (Smith et al. 2015a, 2015b). Streamline termini were assigned to their nearest vertex on the surface of the gray-white matter interface. Streamlines of which either terminus was further than 3 mm from its nearest vertex were discarded. Connectomes were smoothed on the surface using a 20 mm full width at half maximum Gaussian smoothing kernel.

To describe the largest axes of variance in connectivity, we used diffusion map embedding (Coifman and Lafon 2006), a nonlinear dimensionality reduction techniques technique used previously to identify neocortical, hippocampal, and cerebellar functional gradients (Margulies et al. 2016; Guell et al. 2018; Vos de Wael et al. 2018). Gradients were computed and aligned using the BrainSpace toolbox (https://github.com/MICA-MNI/BrainSpace) (Vos de Wael et al. 2020), with the following settings: sparsity thresholding at the 75th percentile, a cosine affinity kernel, diffusion map embedding dimensionality reduction with *α* = 0.5, and automated diffusion time estimation. Gradient computations were performed separately on left and right temporal lobes. Interhemispheric connections were not included in the gradient computation. Left and right gradients were aligned with Procrustes alignment (Langs et al. 2015) as implemented in BrainSpace. To approximate the effect of the alignment, we computed the 5th, 50th, and 95th percentiles of the diagonal elements of the rotation matrices across subjects; lower valued diagonals indicate larger rotations. As the sign of gradients may be freely inverted, rotation matrices were multiplied by the sign of the diagonal element before percentile computation. Eccentricity was computed from the aligned gradients by computing the Euclidean distance to the origin of the manifold space spanned by the first three gradients.

### Statistical Testing

Testing for linear associations between cortical markers and gradients likely leads to biased test statistics due to the spatial autocorrelation of MRI data violating the independence of observations assumption (Alexander-Bloch et al. 2018). Instead, for each statistical test, we generated random datasets with comparable spatial properties. Specifically, we generated random datasets with equivalent spatial autocorrelation as the response variable using Moran spectral randomization with the singleton procedure (Wagner and Dray 2015) as implemented in BrainSpace (Vos de Wael et al. 2020). All linear models were fitted for the original data as well as 1000 corresponding simulated datasets. Presented *P*-values were derived from the percentile rank of the true *F*-statistic in the distribution of *F*-statistics in the simulated data. We further report product moment correlations derived from the empirical data; however, please note that these are only approximate values given the unknown spatial autocorrelation (Burt et al. 2020). Multiple comparisons were corrected for false discovery rate with the Benjamini–Hochberg procedure (Benjamini and Hochberg 1995).

### Tractography Analyses

Connectivity distance, a measure that characterizes the relationship between physical distance and connectivity (Larivière et al. 2020), was computed by thresholding the structural connectivity matrix at the 80th percentile, multiplying each connection by the geodesic distance between their nodes, and averaging all connections for each node. Degree centrality was computed as the column-wise sum of the connectivity matrix. Statistical significance of the association between both degree centrality as well as connectivity distance with the gradients was assessed with Moran spectral randomization (Wagner and Dray 2015).

### BigBrain Gradient

To assess histological properties of the brain we used BigBrain, an ultrahigh-resolution atlas of a single postmortem brain stained for cell bodies (Amunts et al. 2013). Gradients of microstructural profile covariance were computed as described previously (Paquola et al. 2019). In short, 18 equivolumetric surfaces were constructed between the outer and inner cortical surfaces. To reduce partial volume effects, the inner cortical surface was removed. A linear model implemented in SurfStat (Worsley et al. 2009) was used to correct for anterior-to-posterior increases in intensity values (Amunts et al. 2013). Surface vertices were grouped into 1012 parcels which respected the boundaries of the Desikan-Killiany atlas (Desikan et al. 2006; Hong et al. 2017). A microstructural profile covariance matrix was constructed by computing the Pearson’s correlation of every pair of vectors while controlling for the average whole-cortex intensity profile. Gradients were constructed from this matrix using BrainSpace default parameters (90% sparsity, normalized angle kernel, diffusion map embedding, *α* = 0.5, automated diffusion time estimation). To compare structural connectivity gradients to BigBrain gradients, the structural gradients were parcellated using the same parcellation scheme. Moran spectral randomization (Wagner and Dray 2015) was used to test for associations between BigBrain gradient 1 and the structural gradients.

### Functional Predictions

Structural gradients were used to predict canonical resting-state networks published previously [(Yeo et al. 2011); https://surfer.nmr.mgh.harvard.edu/fswiki/CorticalParcellation_Yeo2011]. HCP-Discovery was split into 5-folds of 15 subjects each; for each fold, we performed a multinomial logistic regression with the first three gradients as predictor variables and networks as outcome variables. Beta values derived from the training set were used to predict probabilities of each network in the testing set. Each vertex was assigned to the network with the highest probability. Additionally, we derived beta values from the entire HCP-Discovery dataset and used these to predict functional networks from HCP-Replication and MICs gradients.

To further assess the relationship between structural gradients and edgewise functional connectivity, we used a decision tree with binary splits for regression. Similar to the network prediction, training and testing was performed both with 5-fold cross validation as well as training on HCP-Discovery and testing on the other datasets. Model training was performed with the *fitrtree* function as implemented in MATLAB R2019b with a minimum leaf size of 20, a maximum number of splits of 20, and otherwise default parameters.

### Evolutionary Analyses

We tested for associations between our gradients and two markers of evolutionary change between humans and macaques: functional homology and areal expansion. Both measures were presented in a prior paper (Xu et al. 2020), hence we only provide a short overview here. Functional homology is a measure for the functional similarity of a human brain area with its macaque counterpart. It is computed based on the maximum cosine similarity of functional gradient profiles within a 12-mm search light around the corresponding human/macaque vertices. An areal expansion map shows the relative expansion of human cortex compared to macaques. It is computed by dividing the local area of human cortex by the corresponding area of macaque cortex where correspondence was defined based on functional homology. We tested for associations between these two markers and the structural gradients using Moran spectral randomization (Wagner and Dray 2015).

## Results

Our main analyses were based on 75 unrelated participants of the HCP S900 release (Van Essen et al. 2013), a large-scale open-access neuroimaging dataset comprised of healthy young adults (HCP-Discovery; *n* = 75; age = 29.2 ± 3.6, female = 47). We also replicated all findings in a subset of unrelated participants from HCP, (HCP-Replication; *n* = 75; age = 28.9 ± 4.0, female = 44). For each participant, we mapped structural connectivity of each vertex in the gray–white matter interface of the temporal lobe to the entire cortex using high-resolution tractography (see Methods, for details). To identify structural connectivity gradients, we used nonlinear dimensionality reduction techniques that identify spatial eigenvectors explaining inter-regional variations in structural connectivity (Coifman and Lafon 2006). To assess the reproducibility of our findings, we repeated our analyses on the MICs dataset, a separate dataset of healthy controls who underwent 3 T imaging comparable to the HCP protocol in our center (54 controls, 30.5 ± 7.3 years old, 20 females).

### Multiple Gradients of Structural Connectivity in the Temporal Lobe

**Figure 1:**
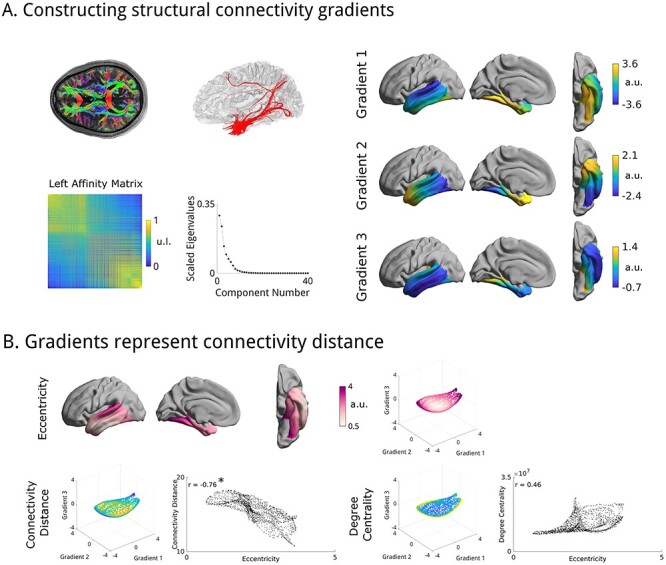
Generation of temporal lobe structural connectivity gradients. (*A*) Streamlines were generated throughout the entire brain and systematically mapped to the cortical surface using nearest neighbor interpolation. We computed the unitless (u.l.) affinity matrix of the connectivity matrix using a cosine similarity kernel and constructed gradients [in arbitrary units (a.u.)] of structural connectivity of the temporal lobe to ipsilateral hemisphere with diffusion map embedding. The first three eigenmodes, sorted by variance explained, described connectivity gradients that were selected for further analyses. (*B*) An eccentricity feature can be computed in this manifold space, by calculating node wise Euclidean distances to the origin of the manifold, this feature was high in posterior medial temporal lobe and the superior temporal gyrus and correlated significantly with connectivity distance (left) but only moderately with degree centrality (right).

In HCP-Discovery, the first three components of temporal cortical gradients collectively explained 67% of variance in temporal lobe structural (Fig. 1*A*). We retained these gradients based on the decrease in size of later eigengaps combined with the practical consideration of allowing for data visualization in 3D space. Furthermore, eccentricity, the main measure used in the remainder of this manuscript, only changed marginally with additional gradients (*r* > 0.99 between any pair of eccentricities derived from between 3 to 10 gradients in all datasets). Findings were similar in the left and right hemispheres (correlations of left/right gradients all *r* > 0.99). We, thus, present only left hemispheric results in the main figures (for right hemisphere results, see Supplementary Fig. S1). Gradient solutions were consistent across the different datasets studied, with absolute correlations between G1–3 of HCP-Discovery with G1–3 of HCP-Replication and MICs exceeding *r* > 0.96.

The first structural connectivity gradient (G1) ran between the superior temporal gyrus and the medial temporal lobe (Fig. 1*A*), the second (G2) along the posterior–anterior axis, and the third (G3) from anterolateral to posteromedial. To determine the connectivity patterns represented by each gradient, we mapped the connectivity of the top/bottom 10% of vertices of each gradient and assessed changes in the spatial distribution of connectivity profiles at the anchors of each gradient. G1 connectivity changes differentiated between visual and parietal connectivity, G2 involved changes from temporal pole and insula to visual/parietal cortex and lateral frontal cortex, and G3 described changes from visual/parietal to lateral temporal and frontal (see Supplementary Fig. S2).

In order to provide a scalar metric in multivariate gradient space and quantify connectome-level differentiation across the cortical mantle, we calculated an eccentricity measure that captures the distance from the origin in the manifold space spanned by the first three gradients (Park, Bethlehem, et al. 2020; Park, Hong, et al. 2020). Low eccentricities were situated in the middle temporal gyrus, while high eccentricity was present in posterior superior temporal and medial temporal regions. To determine the connectivity patterns underlying gradient eccentricity, we performed spatial correlation analyses between eccentricity and topological measures of degree centrality, a measure for the number of connections of a node (Hagmann et al. 2008), and connectivity distance, a measure for the relative strength of a node’s long distance connections (Larivière et al. 2020) (Fig. 1*B*). These measures were chosen as both overall connectedness and the length of connections represent key organizing principles of the human connectome (Hagmann et al. 2008; van den Heuvel and Sporns 2011). Findings were corrected for spatial autocorrelation with Moran Spectral Randomization (Wagner and Dray 2015) implemented in BrainSpace (Vos de Wael et al. 2020), and adjusted for multiple comparisons using a false discovery rate procedure (Benjamini and Hochberg 1995). Gradient eccentricity correlated with connectivity distance in both hemispheres (left/right *r* = −0.76/−0.70, *p*_moran_ < 0.002), but not with degree centrality (left/right *r* = 0.46/0.28, *p*_moran_ < 0.22), indicating that the gradients are partially driven by the strength of long-range connections. Results replicated in all datasets, that is, gradients were bilaterally associated with connectivity distance (left/right HCP-Replication: *r* = −0.75/0.70, *p*_moran_ < 0.002; MICs: *r* = −0.78/0.71, *p*_moran_ < 0.01), but not degree centrality (HCP-Replication: *r* = 0.44/0.27, *p*_moran_ < 0.23, MICs: *r* = 0.40/0.39, *p*_moran_ < 0.19).

To contextualize the gradients through cognitive terminology, we decoded the structural gradients and eccentricity map using Neurosynth, an ad hoc meta-analysis of previous fMRI studies (Yarkoni et al. 2011) (see Supplementary Fig. S3). Both G1, G3, and eccentricity represent axes of sensory functions to self-generated cognitive processes (G1: auditory vs. memory/navigation terms, G3: cognitive vs. auditory terms, eccentricity: cognitive vs. perception terms). G2 differentiated stress/affect related terms from visual/word related terms (e.g., “visual” and “word form” vs. “stress”, “pain,” and “regulation”).

### Microstructural Underpinnings

**Figure 2:**
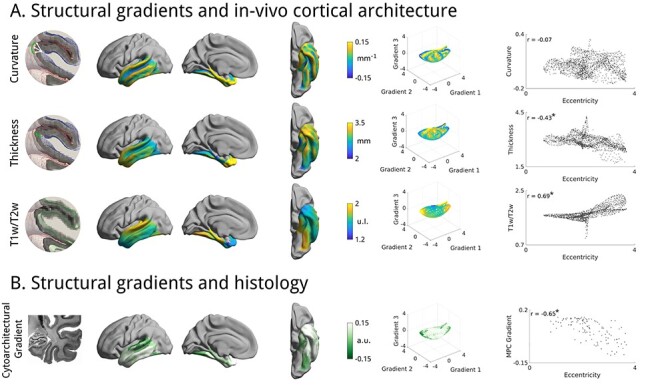
Morphological and microstructural associations. (*A*) We tested for linear relations between manifold eccentricity and curvature, cortical thickness, as well as T1w/T2w intensity. Stars denote significant correlations. (B) We also tested for an association to microstructural profile covariance derived previously from the BigBrain atlas (Amunts et al. 2013; Paquola et al. 2019). Eccentricity was projected to the same parcellation scheme as microstructural profile covariance by taking the mean within each parcel.

Prior research in nonhuman animals has shown inter-regional connectivity is predicted by cytoarchitectural similarity (Barbas 2015), and recent functional MRI work showed correspondence between functional gradients and proxies for intracortical myelin (Huntenburg et al. 2017; Vos de Wael et al. 2018; Paquola et al. 2019; Larivière et al. 2019). Here, we examined the relationship between structural connectivity gradients and in-vivo measures of cortical microstructure. Specifically, we tested for associations of gradient eccentricity and intracortical T1w/T2w intensity, a proxy for myelin (Glasser and Van Essen 2011), and observed a strong association (Fig. 2*A***;** left/right *r* = 0.69/0.78, *p*_moran_ < 0.012). Associations to cortical thickness were only moderate (left/right *r* = −0.43/−0.44, *p*_moran_ < 0.024) and those to curvature did not reach statistical significance (left/right *r* = −0.07/−0.11, It *p*_moran_ < 0.65). Similar findings were seen in HCP-Replication (left/right T1w/T2w *r* = 0.68/0.78, *p*_moran_ < 0.012; cortical thickness *r* = −0.45/−0.44, *p*_moran_ < 0.020; curvature *r* = −0.07/0.12, *p*_moran_ < 0.637) and in the MICs dataset, which used quantitative T1 relaxometry as a myelin proxy (left/right qT1 *r* = −0.36/−0.66 *p*_moran_ < 0.062; cortical thickness *r* = −0.33/−0.31, *p*_moran_ < 0.08; curvature *r* = 0.01/0.03, *p*_moran_ < 0.91). To further study these connectomic associations, we mapped the connectivity of the min/max 10% of each microstructural feature (see Supplementary Fig. S4). We observed more parietal connectivity for areas with lower cortical thickness or higher T1w/T2w intensity and the cytoarchitectonic gradient displayed distinctions in parietal and lateral frontal connectivity.

Next, we evaluated the association between gradients and cortical cytoarchitecture (Fig. 2*B*), capitalizing on the BigBrain dataset, an ultra-high resolution 3D histological reconstruction of a post-mortem human brain (Amunts et al. 2013). We adopted a previously established approach to identify cytoarchitectural gradients (Paquola et al., 2019) and compared the principal cytoarchitectural gradient, which runs from primary sensory to limbic areas, to our in vivo structural connectivity gradients. We found strong associations in both hemispheres (left/right *r* = −0.65/−0.73, *p*_moran_ < 0.002). Again, results were replicated in both HCP-replication (left/right *r* = −0.64/−0.73, *p*_moran_ < 0.002) and MICs (left/right *r* = −0.60/−0.71, *p*_moran_ < 0.028).

### Functional Associations

**Figure 3:**
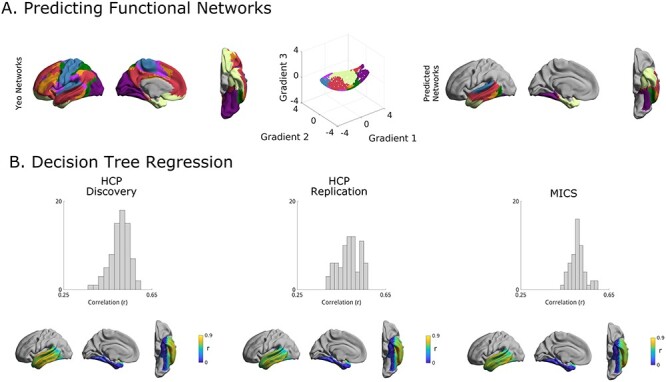
Functional markers of the structural gradients. (*A*) Based on a canonical network parcellation (Yeo et al. 2011), we attempted to predict the functional networks. Performance was high with a Cohen’s kappa of 0.77 ± 0.01 (left) and 0.81 ± 0.01 (right). Predicted networks shown here are the results of one of the five folds. (*B*) Accuracy of left hemispheric decision tree regression. Histograms show the prediction accuracy per subject, as measured by the Pearson’s correlation between empirical and predicted data, of a decision tree regression estimating functional connectivity from structural gradients. HCP-Discovery predictions were trained with a 5-fold cross-validation, the predictions of the other datasets were trained on HCP-Discovery. Cortical surfaces show the Pearson’s correlation between the predicted and empirical functional connectivity for every vertex across subjects. Predictions were especially accurate in lateral temporal regions, and less robust in the medial temporal lobe.

Structural connectivity is ultimately assumed to give rise to functional connectivity (Honey et al. 2009; Deco et al. 2017; Wang et al. 2019; Suárez et al. 2020). As such, we hypothesized that axes of structural connectivity would capture the organization of large-scale functional connectivity. We related the structural connectivity gradients to intrinsic functional community organization, a predominant motif of macroscale neural function (Fig. 3*A*) (Yeo et al. 2011). Using a 5-fold cross validation, we computed group-level structural gradients for the training and testing group. We derived beta values from the training sets with a group-level multinomial logistic regression and used those to predict the layout of the Yeo-Krienen intrinsic functional communities from the group-level testing set. Predictions were accurate and stable (Cohen’s kappa mean ± SD left/right: 0.77 ± 0.01/0.81 ± 0.01). Beta values derived from HCP-Discovery gradients could also accurately predict macroscale functional communities from HCP-Replication (Cohen’s kappa left/right: 0.77/0.82) as well as MICs (Cohen’s kappa left/right: 0.69/0.70).

To further assess how structural connectome gradient features predict regional functional connectivity, we leveraged decision tree regression with Euclidean distances between vertices in gradient space as predictors and edgewise functional connectivity within the temporal lobe as outcome variable. In a 5-fold cross validation trained on group-level folds of HCP-Discovery, gradient manifold distances were predictive of functional connectivity of held-out subjects at the single subject level (Fig. 3*B*; see Supplementary Fig. S5**;** mean ± SD left/right: *r* = 0.50 ± 0.04/0.46 ± 0.05). To assess accuracy of this model at the single subject level a decision tree regression was trained on the entire HCP-Discovery dataset. This model accurately predicted single subject functional connectivity in both HCP-Replication (Fig. 3*B*; left/right *r* = 0.49 ± 0.05/0.43 + 0.05) and MICs (left/right *r* = 0.50 + 0.03/*r* = 0.46 + 0.04). In all datasets, prediction quality was excellent in the lateral temporal lobe but less favorable in the medial temporal lobe. To verify whether the between-subject alignment affected our results, we assessed stability of manifold orientations of the single-subject structural gradients. The first three gradients appeared stable across subjects (5th percentile of the rotation matrices’ diagonal elements > 0.95 across all datasets; see Supplementary Fig. S6).

### Evolutionary Associations

**Figure 4:**
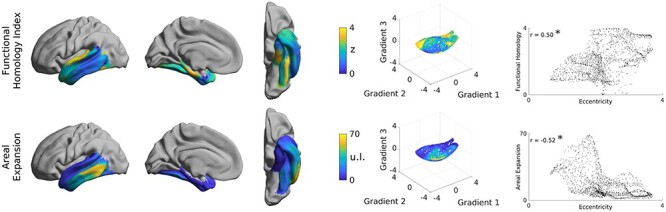
Relationship between the structural gradients and phylogenetic markers. Both functional homology and areal expansion are significantly associated with eccentricity of the first three structural gradients.

Last, we assessed whether eccentricity also relates to measures of phylogenetic changes in the temporal lobe (Fig. 4). We found associations to previously established indices of functional homology (left/right: *r* = 0.50/0.51, *p*_moran_ < 0.04), a measure for similarity of functional organization between human and macaque, and areal expansion (left/right: *r* = −0.52/−0.31, *p*_moran_ < 0.04), a measure for the surface areas increase of human cortex relative to homolog regions in macaques (Xu et al. 2020). At the individual gradient level, both of these markers loaded primarily onto the third gradient (functional homology left/right *r*_g1_ = −0.15/−0.28, *r*_g2_ = 0.20/0.11, *r*_g3_ = 0.60/0.61; areal expansion left/right *r*_g1_ = −0.05/−0.07, *r*_g2_ = −0.49/−0.38, *r*_g3_ = −0.59/−0.49). Results were consistent in both HCP-Replication (functional homology; left/right: *r* = 0.50/0.50, *p*_moran_ < 0.04; areal expansion; left/right: *r* = −0.52/−0.31, *p*_moran_ < 0.04) as well as MICs (functional homology; left/right: *r* = 0.48/0.54, *p*_moran_ < 0.04; areal expansion; left/right: *r* = −0.54/−0.40, *p*_moran_ < 0.03). To further characterize the association between connectivity and these measures, we mapped the connectivity profiles of the min/max 10% of each feature (see Supplementary Fig. S4). Areas with more areal expansion or less functional homology showed more lateral frontal connectivity as well as reduced medial occipital connectivity.

## Discussion

The temporal lobe hosts a diverse array of functional processes implicated in sensory processing, memory, and language abilities (Bonilha et al. 2017; Ralph et al. 2017; Vaz et al. 2019). In addition to its role in healthy brain function and primate evolution, the temporal lobe is among the macroscopic structures most frequently compromised in neurological and neuropsychiatric disorders, including Alzheimer’s disease (Dubois et al. 2010; Stein et al. 2010; Johnson et al. 2016) and drug-resistant epilepsy (Bernasconi et al. 2003; Blümcke et al. 2013; Bernhardt et al. 2015). To comprehensively characterize its substructural organization in humans, our study harnessed manifold learning operating on high resolution diffusion MRI tractography data of the temporal lobe to identify separate, yet partially overlapping axes of its structural connectome embedding. These axes were found to relate to MRI-based measures of intracortical myelination as well as post-mortem cytoarchitecture, supporting potential microcircuit underpinnings of these spatial trends in structural connectivity variations. Supervised machine learning experiments indicated that structural gradients can serve as sensitive, low-dimensional predictors of the functional organization of the temporal lobe. Furthermore, structural connectivity gradients were spatially correlated with previously identified patterns of functional reconfiguration and areal expansion between humans and nonhuman primates, supporting the potential of connectome gradients as axes of evolutionary changes (Xu et al. 2020). Results were reproducible across multiple datasets, indicating generalizability. Collectively, our findings provide robust evidence for an association between structural connectivity, tissue microstructure, and functional motifs of the temporal lobe, which suggests their potential to serve as major organizational axis bridging between its microcircuit layout and macroscale functional role.

Diffusion MRI is currently the only noninvasive method to approximate the course of white matter connections in humans. Based on multishell diffusion acquisitions of the HCP and MICs datasets, we applied constrained spherical deconvolution (Jeurissen et al. 2014) and spherical-deconvolution informed filtering of tractograms (Smith et al. 2015a, 2015b) to estimate streamline weights interconnecting cortical areas. These techniques provide biologically meaningful weights of the modeled streamlines (Smith et al. 2015a, 2015b), and reduce fiber tracking biases (Yeh et al. 2016) as well as partial volume effects (Jeurissen et al. 2014). By propagating each streamline to cortical surface points, rather than to macroscopic parcels, we were able to resolve fine grained changes in temporal lobe connectivity and account for heterogeneity of subregional connectivity. We enhanced this vertex-wise approach with manifold learning techniques that allow for the representation of continuous spatial variations in temporal lobe structural connectivity. Already established by an emerging literature of neuroimaging and network neuroscience studies (Margulies et al. 2016; Huntenburg et al. 2017; Haak et al. 2018; Bajada et al. 2019; Paquola et al. 2019; Blazquez Freches et al. 2020), these techniques model both gradual and overlapping modes of connectivity without reliance on a priori boundaries (Haak and Beckmann 2020). Recapitulating prior work, we found that the temporal lobe is best described by three gradients (Bajada et al. 2019; Blazquez Freches et al. 2020), spanning medio-lateral (G1), anterior–posterior (G2) and anterolateral-posteromedial (G3) axes. Although there have been several reports of asymmetry of the white matter tracts of the temporal lobe, such as greater fiber density and tract volume in the left arcuate fasciculus than the right (for review, see Ocklenburg et al. 2016), the symmetry of the structural gradients identified here suggests gross similarity between the large-scale network embedding of left and right temporal lobes. We then tested for associations with MRI-based measures of curvature, cortical thickness, and intracortical microstructure. In line with our hypotheses and prior work suggesting a close link between internal cortical architecture and structural connectivity (Young 1992; Scannell et al. 1995; Beul et al. 2017; García-Cabezas et al. 2019), we found strong associations between connectome gradients and MRI-derived proxies of cortical myelin. The relationship with cortical thickness and curvature was weaker, suggesting that our cortical connectivity gradients closely reflect intracortical factors and only to a lesser extent mesoscale morphological variations and/or potential biases from sulco-gyral folding (Schilling et al. 2018). A closer link to microstructure was also suggested by harnessing BigBrain derived histology gradients, which describe sensory-fugal trends in internal cortical cytoarchitecture (Amunts et al. 2013; Paquola et al. 2019). Collectively, these findings highlight the close relationship between microstructure and structural connectivity, supporting the extension of the structural model of connectivity to humans in the temporal lobes (Young 1992; Scannell et al. 1995; Beul et al. 2017; García-Cabezas et al. 2019).

Many studies found that structural connectivity may predict functional connectivity by assuming that the strength of functional interactions depends, in part, on the density and efficacy of both direct and indirect structural connections (Honey et al. 2009; Deco et al. 2017; Wang et al. 2019). We hypothesized that the structural gradients, despite their low dimensionality, would still accurately describe functional interactions. Supervised learning approaches with crossvalidation could show that gradient-informed models predicted the spatial layout of previously described intrinsic functional communities in the human brain (Yeo et al. 2011). At a more local scale, gradients could also predict patterns of inter-regional functional connectivity, even when trained and tested on datasets acquired from different scanners. This model was more accurate in lateral regions than in medial regions. One potential cause of this divergence could relate to the increasingly recognized reduction in structure–function coupling in heteromodal and paralimbic regions such as the medial temporal lobe (Paquola et al. 2019; Baum et al. 2020), which would limit the predictability of functional interactions from structural connectivity patterns. Another, not mutually exclusive, option is that the decreased signal-to-noise ratio in the medial temporal lobe reduces the signal fidelity and potentially prediction accuracy. Overall, our results support that eigenmode representations of structural connectivity may potentially underpin intrinsic functional architecture of the human connectome. Such a conclusion is in line with several prior studies in healthy individuals showing that whole-brain structural connectivity gradients shape dynamic signaling at rest (Park et al. 2021) as well as dynamic brain reconfigurations during tasks (Murphy et al. 2019). Furthermore, in the study of brain diseases associated with macroscale dysfunction, connectivity gradients have been used to contextualize changes in brain network architecture (Hong, Vos de Wael, et al. 2019; Larivière et al. 2020; Li et al. 2020; Park, Hong, et al. 2020), supporting their utility to serve as coordinate systems of macroscale functional interactions in healthy and diseased brains.

Cross-species comparisons between humans and nonhuman primates provide a potential window into human uniqueness, and allow studying brain reconfigurations that happened during primate evolution (Krubitzer 2007; Buckner and Krienen 2013). Although a remarkable conservation of macroscale organizational principles between macaques and humans is evident (Glasser et al. 2014; Margulies et al. 2016; Valk et al. 2020), higher association cortices have specifically undergone a striking expansion in relative surface area and potentially increased participation in spatially distributed functional networks (Hill et al. 2010; Buckner and Krienen 2013; Mueller et al. 2013; Patel et al. 2015; Mars et al. 2017). Here, we showed that our structural connectivity gradients spatially align with the pattern of evolutionarily diverging brain areas and areal expansion, an index for relative areal size differences across species (Xu et al. 2020). Areas near the center of the structural manifold were less functionally homologous and have undergone more expansion relative to macaques. This may indicate that evolutionary changes have preferentially occurred along particular fiber tracts including, for example, the arcuate fasciculus which has undergone critical anatomical modifications between nonhuman and human primates (Rilling et al. 2008; Mars et al. 2013; Mars, Eichert, et al. 2018; Ardesch et al. 2019; Eichert et al. 2019, 2020). When taken together with the cognitive terms from the Neurosynth meta-analysis, these results indicate that phylogenetic differences in the temporal lobe are primarily situated along those tracts associated with self-generated cognitive processes.

Theoretical accounts, empirical findings, and gradual changes in research culture have increased the scientific value of replications in neuroscience (Ioannidis 2005; Moonesinghe et al. 2007; Open Science Collaboration 2015). Here, we replicated our findings in two datasets: (1) a set of unrelated young adults derived from the same dataset as the discovery set (HCP-Replication) as well as (2) a separate dataset acquired at the Montreal Neurological Institute (MICs). Even after corrections for both spatial autocorrelation (Wagner and Dray 2015) and multiple comparisons (Benjamini and Hochberg 1995), most findings held across all datasets indicating good reproducibility. We have released all utilized feature data and associated analysis scripts to generate the main figures (https://github.com/MICA-MNI/micaopen), allowing for independent verification of our results and potential follow-up analysis. We hope that these data and associated findings continue to pave the way into studying the important relationship between the microstructure, connectivity, and evolutionary development of the temporal lobe.

## Supplementary Material

2021_Vos-de-Wael_CerCor_R1_sups_bhab149

## Notes

Data were provided, in part, by the HCP, WU-Minn Consortium (Principal Investigators: David Van Essen and Kamil Ugurbil; 1U54MH091657) funded by the 16 NIH Institutes and Centers that support the NIH Blueprint for Neuroscience Research; and by the McDonnell Center for Systems Neuroscience at Washington University. *Conflict of Interest*: None declared.
