# Supplementary material for: Structural Connectivity Gradients of the Temporal Lobe Serve as Multiscale Axes of Brain Organization and Cortical Evolution
Source: Cereb Cortex. 2021 Jun 19;31(11):5151–64. doi: 10.1093/cercor/bhab149 (PMC8491677; doi:10.1093/cercor/bhab149)
Supplement: 2021_Vos-de-Wael_CerCor_R1_sups_bhab149 [file 2021_vos-de-wael_cercor_r1_sups_bhab149.zip › 2021_Vos-de-Wael_CerCor_R1_sups_bhab149.docx]

**Structural connectivity gradients of the temporal lobe serve as multi-scale axes of brain organization and cortical evolution**

Reinder Vos de Wael^1^, Jessica Royer^1^, Shahin Tavakol^1^, Yezhou Wang^1^, Casey Paquola^1^, Oualid Benkarim^1^, Nicole Eichert^2^, Sara Larivière^1^, Ting Xu^3^, Bratislav Misic^1^, Jonathan Smallwood^4^, Sofie L. Valk^5^, Boris C. Bernhardt^1^

*^1^ Montreal Neurological Institute, McGill University, Montreal, Quebec, Canada; ^2^ University of Oxford, Oxford, United Kingdom; ^3^ Center for the Developing Brain, Child Mind Institute, New York, NY, USA; ^4^ Queens University, Kingston, Ontario, Canada; ^5^ Max Planck Institute for Human Cognitive and Brain Sciences, Leipzig, Germany.*

**Supplementary Materials**

Supplementary Figures

**
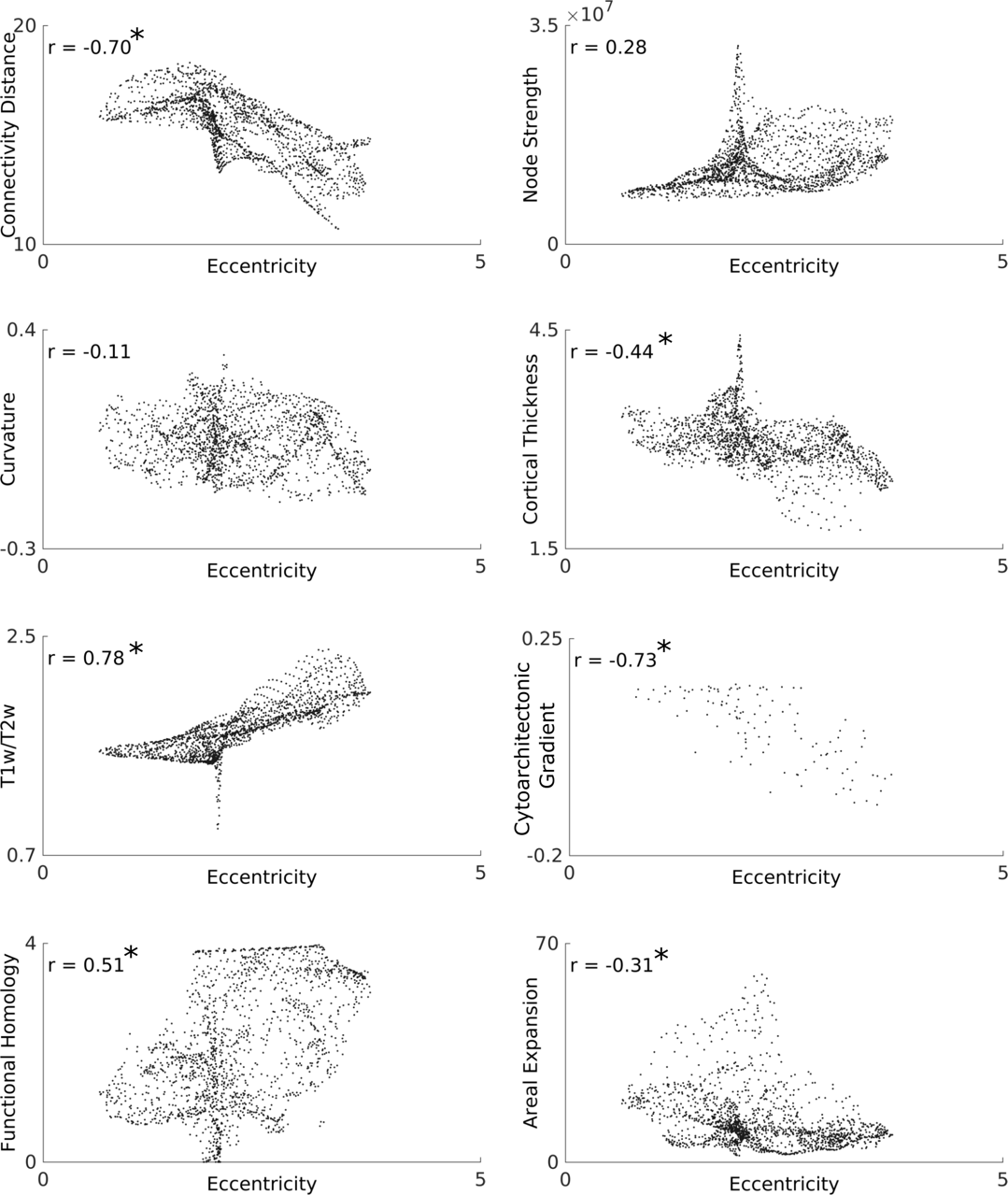
**

**Supplementary Fig. 1** Main right hemispheric results of *HCP-Discovery*. Stars denote significant results (p_moran_<0.05).

*
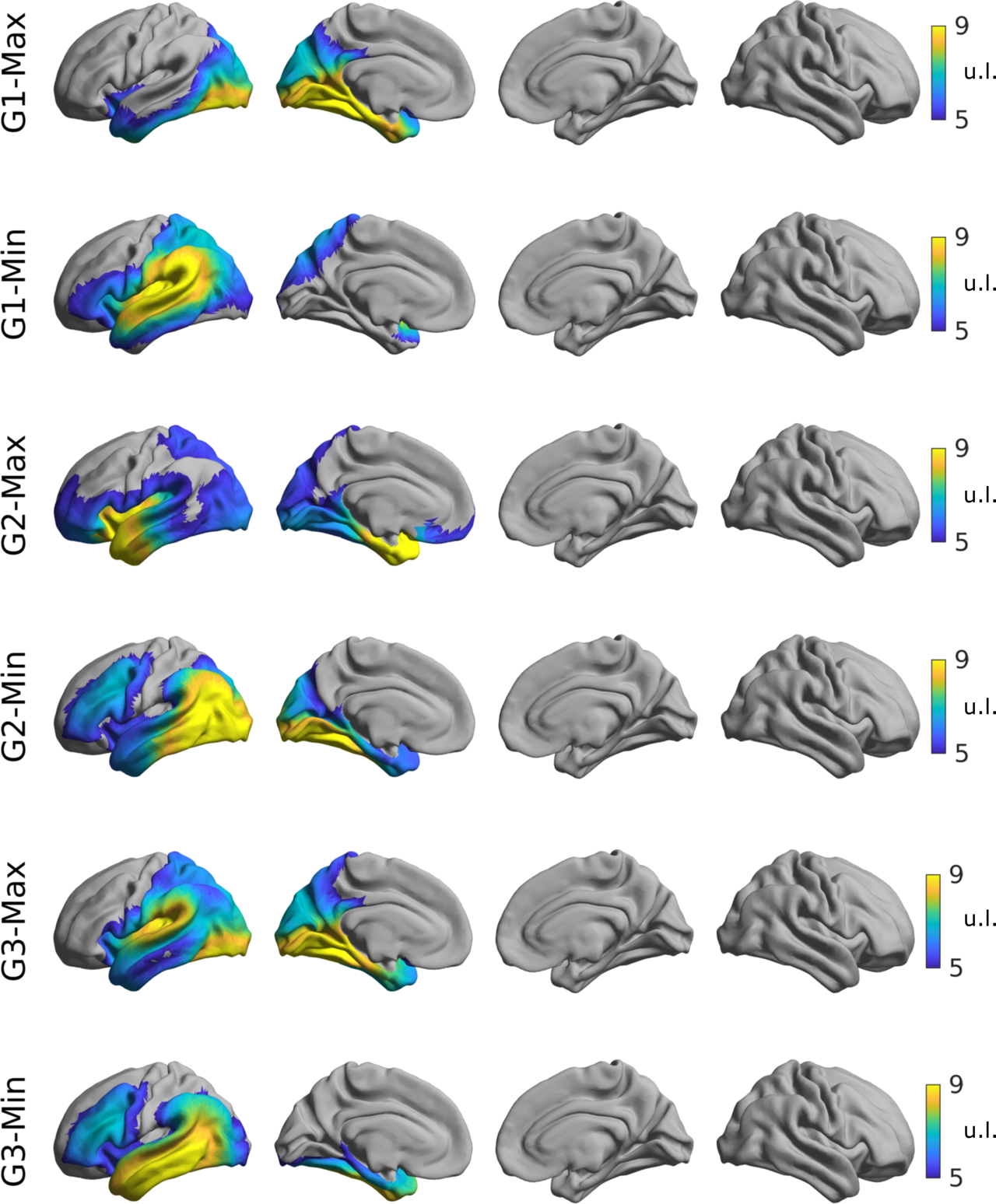
*

**Supplementary Fig. 2.** Connectivity profiles of the top (G-max) bottom (G-min) 10% of the left-hemispheric gradients. Data were log-transformed for visualization purposes only. Colorbars are unitless (u.l.).

**
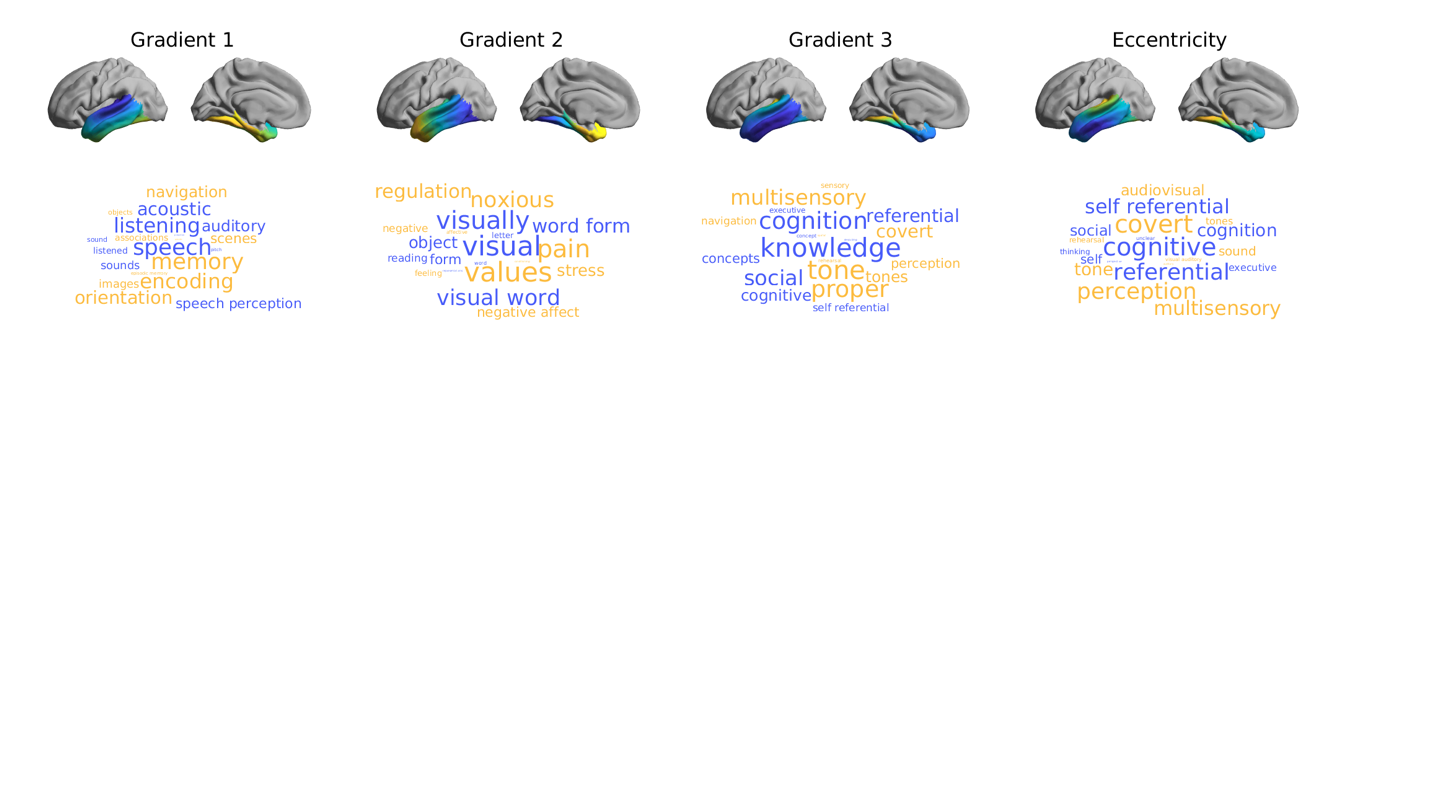
Supplementary Fig 3.** Associations between gradient eccentricity and meta-analytic cognitive terms derived from Neurosynth. Terms in blue are associated with the lower (blue) end of the gradients and yellow terms with the higher (yellow end).


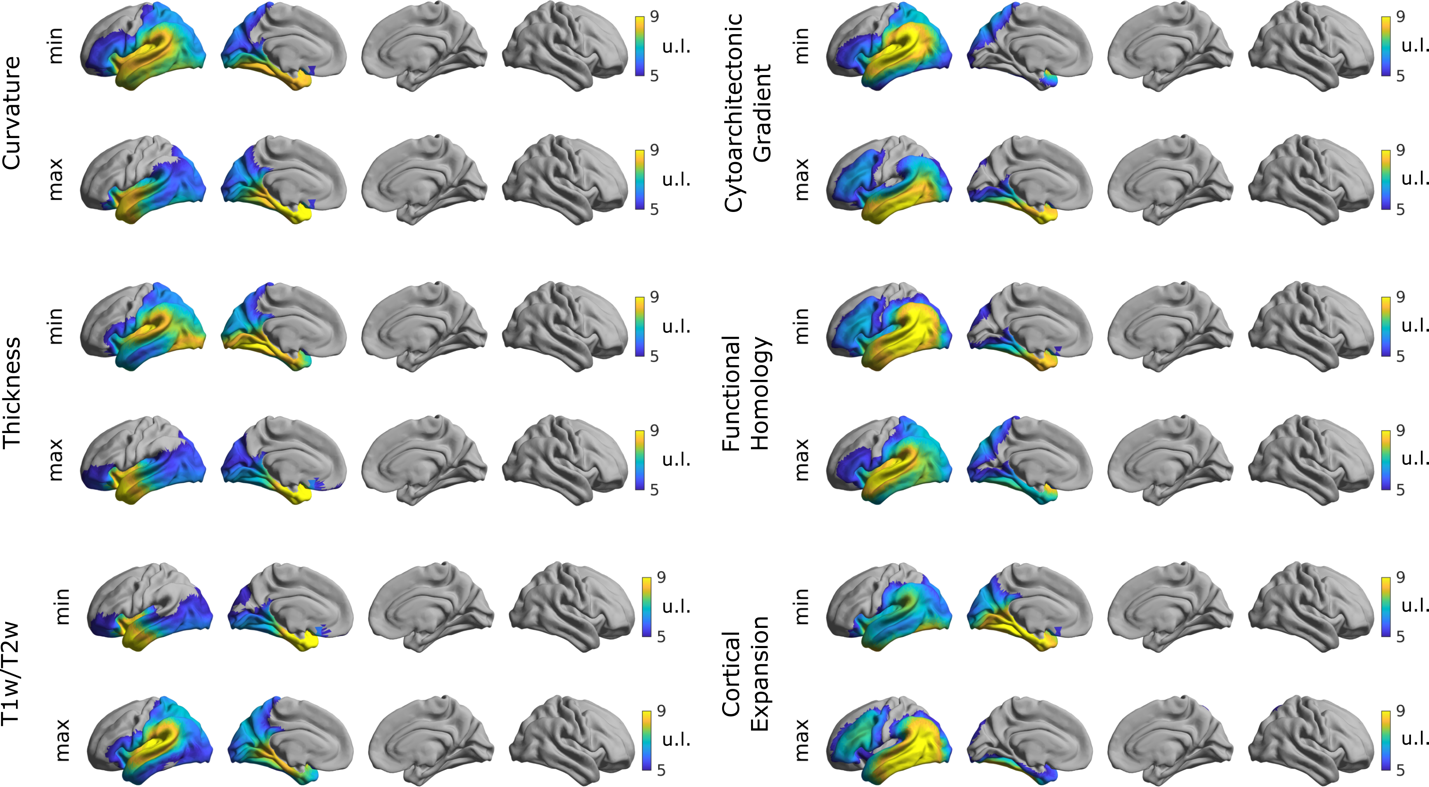


**Supplementary Fig. 4.** Mean left-hemispheric connectivity profiles of the 0-10^th^ percentiles (min) and 90-100^th^ percentiles (max) of each structural and phylogenetic measures. Data were log-transformed for visualization purposes only. Colorbars are unitless (u.l.).

**
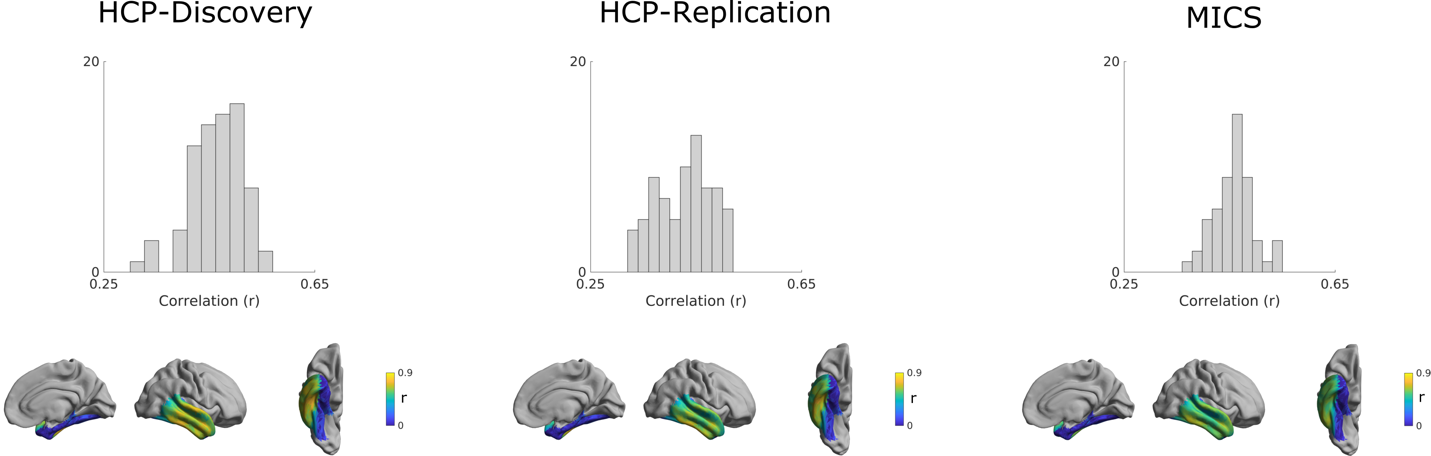
Supplementary Fig. 5.** Accuracy of right hemispheric decision tree regression. Histograms show the prediction accuracy per subject, as measured by the Pearson correlation between empirical and predicted data. HCP-Discovery predictions were trained with a 5-fold cross-validation, the predictions of the other datasets were trained on HCP-Discovery. Cortical surfaces show the Pearson’s correlation between the predicted and empirical functional connectivity for every vertex across subjects.

**
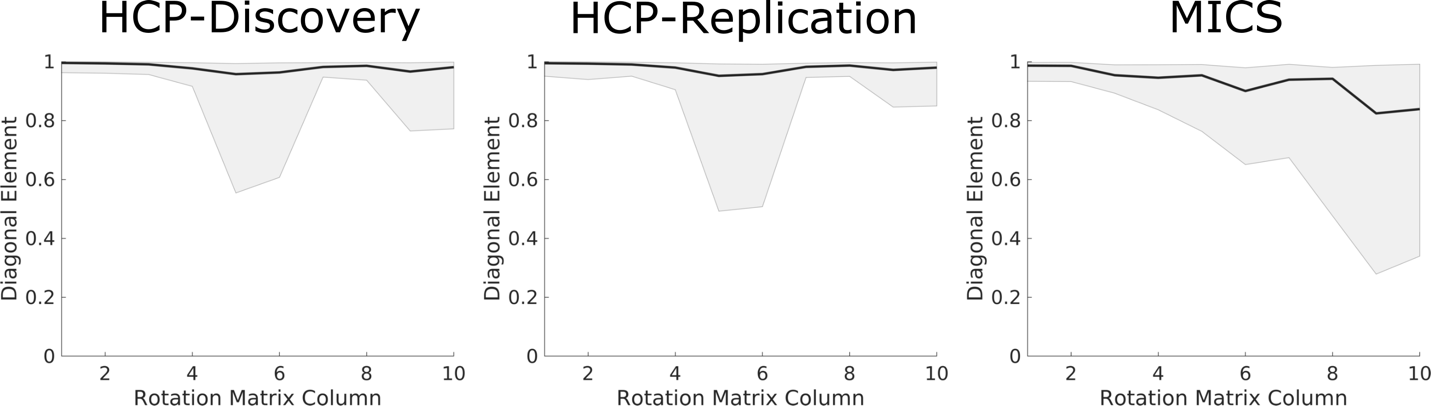
Supplementary Fig. 6.** Stability of gradient manifold orientation across subject. The solid line shows the 50^th^ percentile of the diagonal elements of the rotation matrix across subjects. Light gray areas show the 5^th^ and 95^th^ percentiles. Values close to 1 denote gradients that are relatively unaffected by Procrustes alignment.
